# Assessing Biological Mortality Bias From Deciduous Tooth Emergence

**DOI:** 10.1002/ajpa.70202

**Published:** 2026-01-10

**Authors:** Darryl J. Holman, Chris J. Dolinski

**Affiliations:** ^1^ Department of Anthropology, Center for Studies in Demography and Ecology, Center for Statistics and the Social Sciences University of Washington Seattle Washington USA; ^2^ Department of Anthropology University of Washington Seattle Washington USA

**Keywords:** dental eruption, paleodemography, paleoepidemiology, selective mortality

## Abstract

**Objectives:**

Traits found in a skeletal sample are frequently used to infer qualities of the living population from which the skeletons were drawn. However, traits observed in a mortality sample may exhibit biological mortality bias in that they may not accurately represent the same traits in the living sample. The purpose of this research was to assess biological mortality bias in deciduous tooth emergence, a trait that is used to estimate chronological age in skeletal samples.

**Materials and Methods:**

Data on clinical tooth emergence were collected from longitudinal studies of Javanese, Guatemalan, and Bangladeshi children that included a *living sample* (those who survived through the study) and a *mortality sample* (those who died during the study). Parametric hazards analysis was used to test for differences in the timing of tooth emergence between the living and mortality samples.

**Results:**

There were no significant differences between the living and mortality samples for Bangladesh and Java, although there was a trend toward delayed emergence in the Bangladesh mortality sample. The Guatemalan mortality sample exhibited advanced emergence of the posterior dentition for the mortality sample. No evidence of biological mortality bias was found in pooled analyses of the Bangladesh and Java samples or pooled analyses of all three samples.

**Discussion:**

We found limited evidence that deciduous tooth emergence in a mortality sample (e.g., a skeletal series) would differ from the timing of emergence in the living population from which the mortality sample was drawn.

Paleodemographers and paleoepidemiologists use skeletal samples to investigate the health, growth, and demographic characteristics of past populations (Boldsen and Milner 2012; Milner et al. 2008). An implicit assumption in most investigations is that characteristics or traits assessed in the skeletal sample are representative of the living population from which they were drawn. However, the validity of using a skeletal sample to represent the living population has long been questioned (Armelagos et al. 1972; Cook 1981; Cook and Buikstra 1979; Johnston 1968; Merchant and Ubelaker 1977; Wood et al. 1992). Individuals at a particular age in a cemetery sample have been “selected” through death. As a result, they may be selected for characteristics that increase the risk of mortality, and may systematically differ in those characteristics from same‐aged individuals who died later in life. Likewise, factors that contributed to the deaths of individuals also affected the characteristics of interest. For example, the mortality sample may have had lower food availability, a higher disease load, or may have differed from the living sample in physiological characteristics like susceptibility to disease or the ability to fight infection.

The more general and interrelated phenomena of selective mortality and frailty differences among individuals (Wood et al. 1992) may produce what has been termed biological mortality bias, defined as a physiological and morphological difference between individuals of a given age who die and those who survive (Saunders and Hoppa 1993).^1^ Biological mortality bias is an outcome of selective mortality that arises when there is a correlation between risk factors for mortality and the expression of skeletal traits like long bone growth or tooth emergence. It can be realized at two stages.

First, there can be mortality bias in the target trait of interest. For example, long‐bone growth is frequently used to assess the health of a population based on a series of sub‐adult skeletons (Buikstra and Cook 1980; Johnston and Zimmer 1989; Saunders 1992). Mortality bias in age‐specific long bone length would exist if, on average, children who die prior to completing their long‐bone growth are developmentally behind same‐aged children who survive to maturity (Spake and Cardoso 2019). Hence, inferences about the underlying population would unwittingly reflect this bias toward slower growth when sub‐adult skeletal samples are used to reflect the broader population (Cook 1979).

Biological mortality bias can also arise when skeletal traits are used to estimate the age of a skeletal sample. Growth traits must be standardized relative to chronological age in order to compare the trait in a target sample to the same trait in other populations. In the absence of vital records, age is indirectly estimated from skeletal traits. Dental development shows a high correlation to chronological age and is therefore one of the most accurate macro‐indicators used in skeletal age estimation (Hotz 1959; Lewis and Garn 1960; Krogman 1968; Demirjian et al. 1973; Saunders et al. 1993; Shackelford et al. 2012; Šešelj et al. 2019). Health can then be assessed by other developmental markers like long bone growth for a given dental age. If dental development is adversely affected in the mortality sample, then our inference of health is based on biased estimates of chronological age (Spake et al. 2021).

This paper focuses on this second aspect of biological mortality bias. We examine whether a mortality bias exists in deciduous tooth emergence. Emergence of the deciduous teeth is one type of skeletal trait that is used for age estimation of children from birth to about 3 years (Townsend and Hammel 1990; AlQahtani et al. 2010). The age at emergence for deciduous teeth is considered to be robust to moderate environmental insults, malnutrition, and disease (Jelliffe and Jelliffe 1973; McGregor et al. 1968; Reddy 1981; Saleemi et al. 1993; Shuper et al. 1985), so that emergence has been widely used to age children in populations without written age documentation and as a marker of growth and development for infants and young children (Jelliffe and Jelliffe 1973). Some more recent investigations suggest that nutritional stress (Alvarez 1995; Bastos et al. 2007; Gaur and Kumar 2012), poor health (Alvarez 1995; Holman and Yamaguchi 2005), and famine exposure (Holman and Basu, n.d.) can significantly delay tooth emergence, while high birth weight and excess nutrition can accelerate dental growth (Möhlhenrich et al. 2023; Strumpf et al. 2023). This suggests a need for reference samples drawn from environments with similar levels of nutrition and diseases as the target skeletal sample. Indeed, environmental differences likely contribute to differences in the timing and pattern of tooth eruption found among populations (Holman and Jones 1998; Muthu et al. 2024).

Whether or not tooth emergence is buffered against environmental stresses like under nutrition or high disease burden, the possibility still remains that individuals who are biologically more frail may show a different pattern of dental emergence and be at a higher risk of mortality (Wood et al. 1992). This biological mortality hypothesis is difficult to test directly because high‐quality measures of biological frailty do not exist for skeletal samples (Spake and Cardoso 2019). Instead, indirect methods must be used. Saunders and Hoppa (1993) suggest that this problem can be investigated with historic cemeteries where comparisons can be made between skeletons and documents (e.g., Saunders et al. 1993; Lampl and Johnston 1996). Spake et al. (2021) segregated mortality samples by cause of death, assuming those dying of natural causes approximate a frailer mortality group and those dying of accidental causes are a less frail group that approximates survivors. An alternative way of investigating the issue, suggested by Johnston (1968), is by examining growth and development in living populations. A version of this was done in Stull et al.'s (2021) investigation by using radiographic imaging. Researchers generated living and mortality samples from X‐rays and CT scans of age‐specific differences in long bone growth and dental development.

In this paper, we use this second approach, examining the timing of tooth emergence in three longitudinal investigations of growth and development in children. If there is a biological mortality bias in deciduous tooth emergence, it would appear through differences in the timing of deciduous dental emergence between the subset of children who die during a prospective study and those who do not. We examined dental emergence in Guatemalan, Bangladeshi, and Javanese children. Parametric event history analysis was used to assess differences in emergence during the study period between children who died (the mortality sample) and those who survived to the end of the study (the living sample).

The three samples investigated in this paper provide for something of a natural experiment to examine health effects on biological mortality bias. Although all three samples are in non‐industrial settings, each differs with respect to health and nutrition. The Bangladeshi and Javanese samples are characterized by chronic undernutrition and a lack of modern health care. At the start of the Bangladesh study a severe famine occurred that added acute nutritional stress to a population already experiencing chronic nutritional stress. The Guatemalan sample is from an intervention study that examined the effect of protein and calorie supplementation on child development; children received limited modern health care as well. We do not mean to imply that any of these populations are representative of prehistoric or particular cemetery populations. Rather, these studies allow us to assess whether a mortality bias can be detected in deciduous tooth emergence over a range of ecological contexts in contemporary humans that may inform bioarchaeological inquiry.

Although we examine clinical emergence as the trait of interest, alveolar emergence is used in skeletal samples. It is unlikely that biological mortality bias would manifest differently between the two, as they are both specific landmarks for the same underlying eruption process (Konigsberg and Holman 1999). If biological mortality bias occurs in clinical emergence assessed in living populations (with a mortality sub‐sample), it seems likely that the trait would exhibit biological mortality bias in skeletal samples as well.

## 1 Materials and Methods

Data were collected from longitudinal studies of deciduous tooth emergence in Javanese, Guatemalan, and Bangladeshi children. In each study, clinical emergence was assessed over periodic intervals, so that observations are either interval censored or right censored. We refer to children who survived through the study as the *living sample* and children who died during the study as the *mortality sample*. Parametric hazards analysis was used to estimate parameters for the distributions of times to tooth emergence, modeling the effect of dying during the study as a covariate. In each study, a tooth was considered emerged if any part of the tooth had, upon direct inspection of the mouth, pierced the gum line (clinical emergence). Records for left dentitions were used for all analyses, as the left and right dentitions are highly correlated (Holman and Jones 1991).

### 1.1 Samples

Designs and protocols for the three studies were broadly similar, only differing in minor characteristics such as age at recruitment, number and timing of visits, study length, and dropout patterns. In all three studies, each child's date of birth was known.

*Bangladeshi children*. These data were collected as part of the Meheran Growth and Development Study conducted from October 1973 to January 1978 in the rural village of Meheran, located in Matlab thana (a rural administrative district similar to a county in the United States) about 50 km south‐east of Dhaka (ICDDR,B 1990; Khan et al. 1979, 1981). Most of the children were recruited at birth but some children were enrolled at later ages. Dental records were prospectively collected on 397 children by trained personnel. Clinical examinations were conducted monthly for the first year of the study and quarterly for each year thereafter. Previous analyses of the dental data are given elsewhere (Khan et al. 1981; Holman and Jones 1998, 2003). At the time, health care in Matlab was typically administered by unlicensed village practitioners using traditional remedies (Bhuiya and Streatfield 1992). Over the course of the study, 38 children died.

In early 1974, Bangladesh experienced extensive flooding followed by a severe famine through September 1975 (Hernández‐Julián et al. 2014). During this time, death rates increased, birth rates fell sharply, and nutritional status declined (Langsten and Chowdhury 1984; Bairagi 1986). About 1.5 million Bangladeshis died in the famine (Razzaque 1989). The infant mortality rate in Matlab rose from 110.7 per thousand in 1966 to 167.2 per thousand in 1974 before declining to pre‐famine levels two years later (Begum 1983).

*Guatemalan children*. These data are from the Institute of Nutrition of Central America and Panama (INCAP) longitudinal study of nutrition and mental development carried out from 1968 to 1977 in four Spanish‐speaking Guatemalan farming villages in the department of El Progreso, northeast of Guatemala City in a dry, mountainous area. Most households participated in agricultural production as tenant farmers or small landowners (Habicht and Martorell 1992).

Mothers and children were examined every 3 months from child's birth to 2 years of age and every 6 months thereafter. The sample includes dental records for 1277 children. Most children were enrolled at birth, although some older children were recruited into the study. A description of the dental data as well as analyses of emergence can be found in Delgado et al. (1975) and Holman and Jones (1998, 2003). Over the course of the study, 39 children died.

Nutritional supplements were provided freely to all residents: two villages received a protein supplement and two received a calorie drink. Over the course of the study, health care was provided by a physician and through clinics employing paramedic personnel. Children who showed signs of malnutrition (marasmus or kwashiorkor) during the study were provided additional nutritional interventions (Habicht and Martorell 1992; Read and Habicht 1993), resulting in considerable improvements in health and nutrition. Infant mortality was reduced from 139 per 1000 births prior to 1969 to 55 per 1000 births by 1972, while national death rates in Guatemala remained constant (Rose et al. 1992).

*Javanese children*. These data are from the Ngaglik project, a study of breastfeeding behavior and birth interval dynamics, carried out from 1976 to 1978 in Central Java, Indonesia (Ngaglik Study Team 1976; Hull 1978, 1979). Descriptions and analyses of the dental data can be found in Hull (1983) and Holman and Jones (1991, 1998, 2003). A cohort of 510 women was enrolled in the study, based on a recent delivery or an ongoing pregnancy. Their infants were recruited into the study from birth to 6 months of age. Survey personnel visited the women and infants every 35 days for up to 2.5 years. Dental records from 468 children were available for analyses, of which 48 died during the study.

Households that participated in the study spanned a narrow socioeconomic range from low to moderate income as assessed by an economic survey (Hull 1978, 1983). Most households were involved in rural agriculture, and the mothers regularly engaged in economic activities like farming, farm labor, and small‐scale trading. The staple food was rice, supplemented by cassava for part of the year. Health care for the children typically consisted of homemade herbal medicines and other traditional cures. Traditional healers were rarely consulted, and then only after efforts within the household failed (Hull 1979).

### 1.2 Statistical Methods

The statistical methods are fully described elsewhere (Holman and Jones 1998; Holman and Yamaguchi 2005) and are summarized here. Parametric event history analysis was used to assess differences in deciduous tooth emergence between the living sample and the mortality sample during the study period. The distribution of tooth emergence is modeled as a lognormal distribution, *f*(*t*|*a*, *b*), with two intrinsic parameters, *a* and *b*, and survival function *S*(*t*|*a*, *b*).^2^ The *a* parameter is the shape parameter, and the *b* parameter is the scale parameter. Children's ages at emergence (*t*) are taken from eight months before birth, corresponding to the time just prior to when the dental lamina is formed (Ten Cate 1998), as suggested by Kihlberg and Koski (1954) and Hayes and Mantel (1958), although we report mean emergence times from birth.

All observations are either interval censored or right‐censored. We define the last age at which the child was observed without emergence as *t*_*u*_ and the first age at which emergence is observed as *t*_*e*_. Emergence occurred at some unknown age (*t*) in the half‐closed interval [*t*_*u*_, *t*_*e*_). For observations that are right censored, *t*_*e*_ is ∞.

Covariates included in all analyses were *died* (no = 0, yes = 1) and *sex* of the child (girl = 0, boy = 1). For the pooled analysis, covariates denoting population membership were included: *Bangladesh*, *Java*, or *Guatemala*, coded as 1 for membership and 0 otherwise. For the pooled analyses of all three samples, *Guatemala* = 1 was the omitted reference category. For analyses of the pooled Bangladesh and Java studies, *Java* = 1 was the omitted reference category. We modeled covariates as affecting the hazard of emergence using a standard parametric proportional hazards specification $S\left(t|a,b,\beta \right)=S{\left(t,a,b\right)}^{\exp\left({x_{i}}^{\prime }\beta \right)}$ where ${x_{i}}^{\prime }\beta$ is the vector of products of covariates and *β* coefficients to be estimated. The likelihood function for *N* interval or right censored observations is

(1) $$L=\prod_{i=1}^{N}\left[S{\left(t_{u_{i}}|a,b\right)}^{\exp\left({x_{i}}^{\prime }\beta \right)}-S{\left(t_{e_{i}}|a,b\right)}^{\exp\left({x_{i}}^{\prime }\beta \right)}\right].$$

Estimates of the parameters *a*, *b*, and covariate parameters (*β*_sex_, *β*_died_ and for pooled analyses, *β*_Bangladesh_, *β*_Java_) were found by maximizing likelihood (1).

Estimates of parameter uncertainty were found by conventional bootstrap resampling. For each tooth in each sample, 2001 bootstrap resampled datasets were created and parameters were estimated from each data set. The median estimate was found as the 1001st ordered estimate, and the central 95% mass of the parameter estimates was taken as the 95% confidence intervals about the median estimate.

For a given set of parameter estimates, means and other summary statistics can be found numerically. For example, for a male who has died during the study, the mean emergence age is computed from the two intrinsic and two covariate parameter estimates as follows:

(2) $$\hat{\mu }=\int_{0}^{\infty }S{\left(t|\hat{a},\hat{b}\right)}^{\exp\left({\hat{\beta }}_{\mathrm{sex}}\times 1+{\hat{\beta }}_{\text{died}}\times 1\right)}\mathrm{dt}$$

An analysis of statistical power was used to assess the ability to detect a difference between the living and mortality samples for each tooth. The sampling distribution estimated for the *β*_died_ parameter is used to find the sample size needed to have an 80% probability of detecting a difference between the living and mortality samples for a range of effect sizes from one to six months of delayed emergence. We start by numerically finding values of *β*_died_ parameters that yield delays in the mortality sample of 1 month through 6 months; these are *β*_1mo_, …, *β*_6mo_. The standard deviation of bootstrapped estimates for *β*_died_, ${\hat{\sigma }}_{\beta_{\text{died}}}$, is taken as the sampling distribution for the observed effective sample sizes (*N*_eff_) for each tooth. The effective sizes for the three data sets analyzed here are found in Holman and Jones (1998). To have an 80% probability of detecting a one‐month delay in the mortality sample, the required effective sample size (*N*_1mo_) is estimated as:

(3) $$N_{1\mathrm{mo}}=N_{\mathrm{eff}}{\left(\frac{{\hat{\sigma }}_{\beta_{\text{died}}}}{\left|{\hat{\beta }}_{1\mathrm{mo}}\right|/\left(z_{\alpha }+z_{\beta }\right)}\right)}^{2}$$

where *z*_*α*_ is 1.96 for a 95% confidence interval, and *z*_*β*_ is 0.84 for a power of 80%. The numerator of (3) is the estimated standard error for the observed effective sample size (*N*_eff_). The denominator provides an estimate of the standard error needed for the desired effect size (1 month, in this example) and power. The square of that ratio scales the effective sample size to the size needed to detect that effect for the specified power. We used this method to numerically find the effect size for the effective sample sizes observed for each tooth in the study.

## 2 Results

Table 1 through Table 3 show the parameter estimates for the Bangladeshi, Guatemalan, and Javanese samples, along with the mean emergence ages for the living and mortality subsamples and numbers of right‐censored observations for both the living subsample and the mortality subsample. For the Javanese upper and lower second molars (Table 3), all observations for the mortality subsample were right‐censored, so it was not possible to find estimates for the *β*_died_ parameter. The study was only 2.5 years long and given the small size of the mortality subsample (*N* = 48), it is not surprising that all children in the mortality subsample died prior to emergence of the second molars. The statistical methods, however, allow us to make use of both right‐censored and interval‐censored observations for all the other teeth.

**TABLE 1:** Parameter estimates, mean emergence times, percent censored observations, and detectable effect sizes for the Bangladeshi sample.

Tooth	Median parameter estimates (95% central confidence interval)				Mean emergence age (months)		Percent right censored		*N*_eff_	*E*_mo_
	*a*	*b*	*β*_sex_	*β*_died_	Alive	Died	Alive	Died		
i^1^	2.92 (2.88, 2.97)	0.145 (0.109, 0.179)	−0.231 (−0.530, 0.043)	−0.516 (−1.12, 0.288)	11.8	13.2	19.5	45.5	197	4.5
i^2^	3.00 (2.96, 3.06)	0.146 (0.116, 0.176)	−0.277 (−0.539, −0.018)	−0.694 (−1.41, 0.199)	13.6	16.2	23.6	60.6	178	5.5
c^1^	3.30 (3.27, 3.34)	0.102 (0.083, 0.124)	−0.367 (−0.644, −0.109)	−0.131 (−0.848, 0.574)	21.0	21.2	36.8	69.7	138	4.3
m^1^	3.16 (3.12, 3.21)	0.109 (0.088, 0.134)	−0.040 (−0.317, 0.250)	−0.600 (−1.37, 0.237)	15.9	17.4	29.7	63.6	152	4.8
m^2^	3.57 (3.51, 3.64)	0.156 (0.117, 0.199)	0.103 (−0.173, 0.374)	−0.053 (−0.508, 0.367)	27.0	27.2	40.9	72.7	75	5.1
i_1_	2.86 (2.83, 2.91)	0.129 (0.109, 0.151)	−0.210 (−0.434, 0.045)	−0.722 (−1.42, 0.189)	10.4	12.3	17.0	45.5	201	4.5
i_2_	3.15 (3.10, 3.21)	0.159 (0.132, 0.188)	−0.077 (−0.328, 0.150)	−0.563 (−1.24, 0.131)	16.1	18.2	29.4	66.7	182	6
c_1_	3.34 (3.29, 3.39)	0.116 (0.092, 0.140)	−0.410 (−0.723, −0.084)	−0.008 (−0.583, 0.594)	22.5	22.4	39.0	69.7	106	4.1
m_1_	3.19 (3.15, 3.25)	0.101 (0.088, 0.116)	−0.346 (−0.669, 0.089)	−0.769 (−1.54, 0.202)	17.9	19.9	32.1	69.7	89	5.3
m_2_	3.60 (3.54, 3.68)	0.145 (0.107, 0.190)	0.262 (−0.033, 0.571)	0.022 (−0.679, 0.829)	27.3	27.2	41.5	72.7	60	9.8

*Note:* A sample of 397 children of whom 38 died during the study. Median of 2001 bootstrapped replicates; 95% CIs for *β* parameters that include zero are not significant. *N*_eff_ is the effective sample size that contributed to estimates; *E*_mo_ is the effect size (months) that would have an 80% probability of being measured as statistically significant for the given *N*_eff_.

For the Bangladesh sample (Table 1) and the Javanese sample (Table 3), the 95% central confidence interval for the *β*_died_ parameter of each tooth includes zero. In other words, there is no statistically significant evidence of mortality bias for any tooth in the two populations with the greatest degree of nutritional stress. The small size of the Bangladesh study provided little power to detect differences between the living and mortality subsamples under about 4 months (Table 1). The Javanese sample had larger effective sample sizes for the anterior dentition, but other than i^1^ and i^2^ there was little power to detect differences between the living and mortality subsamples of under 2.5 months (Table 3).

In the Guatemala sample, the *β*_died_ parameters for c_1_, m^1^, m_1_, and m^2^ were significantly greater than zero (Table 2). This indicates that children who died during the study were advanced in emergence timing for these teeth relative to children who survived to the end of the study. The effective sample sizes from the Guatemala study were large enough to detect differences between the living and mortality subsample of about 2 months for all but the second molars.

**TABLE 2:** Parameter estimates, mean emergence times, percent censored observations, and detectible effect sizes for the Guatemalan sample.

Tooth	Median parameter estimates (95% central confidence interval)				Mean emergence age (months)		Percent right censored		*N*_eff_	*E*_mo_
	*a*	*b*	*β*_sex_	*β*_died_	Alive	Died	Alive	Died		
i^1^	2.88 (2.86, 2.90)	0.114 (0.106, 0.124)	−0.167 (−0.310, −0.030)	0.031 (−0.355, 0.418)	10.6	10.5	7.6	55.3	282	1.5
i^2^	2.92 (2.90, 2.94)	0.122 (0.115, 0.130)	−0.136 (−0.258, −0.004)	−0.086 (−0.505, 0.335)	11.3	11.5	8.6	57.9	348	1.8
c^1^	3.27 (3.26, 3.29)	0.101 (0.094, 0.107)	−0.190 (−0.297, −0.097)	0.412 (−0.025, 0.794)	19.3	18.2	14.3	71.1	477	2.1
m^1^	3.17 (3.15, 3.19)	0.095 (0.089, 0.101)	−0.055 (−0.226, 0.110)	0.462 (0.229, 0.827)	16.1	15.3	12.4	63.2	269	1.3
m^2^	3.56 (3.55, 3.58)	0.100 (0.095, 0.105)	−0.070 (−0.177, 0.038)	0.637 (0.182, 1.358)	28.0	25.8	21.2	86.8	461	4.2
i_1_	2.77 (2.75, 2.79)	0.137 (0.127, 0.149)	−0.034 (−0.165, 0.183)	−0.268 (−0.675, 0.142)	8.3	8.8	5.4	47.4	386	1.7
i_2_	3.06 (3.04, 3.08)	0.136 (0.127, 0.144)	−0.075 (−0.182, 0.034)	0.092 (−0.184, 0.386)	13.8	13.3	10.9	57.9	449	1.5
c_1_	3.31 (3.30, 3.33)	0.110 (0.102, 0.116)	−0.101 (−0.204, 0.010)	0.407 (0.030, 0.732)	20.1	19.1	14.8	73.7	484	1.9
m_1_	3.23 (3.20, 3.25)	0.125 (0.097, 0.127)	0.122 (−0.066, 0.227)	0.446 (0.156, 0.800)	17.4	16.3	12.9	63.2	331	1.9
m_2_	3.55 (3.53, 3.57)	0.115 (0.105, 0.126)	−0.027 (−0.170, 0.138)	0.262 (−0.114, 0.604)	27.0	26.3	20.8	86.8	255	2.8

*Note:* A sample of 1271 children and 39 who died during the study. Median of 2001 bootstrapped replicates; 95% CIs for *β* parameters that include zero are not significant. *N*_eff_ is the effective sample size that contributed to estimates; *E*_mo_ is the effect size (months) that would have an 80% probability of being measured as statistically significant for the given *N*_eff_.

**FIGURE 1:**
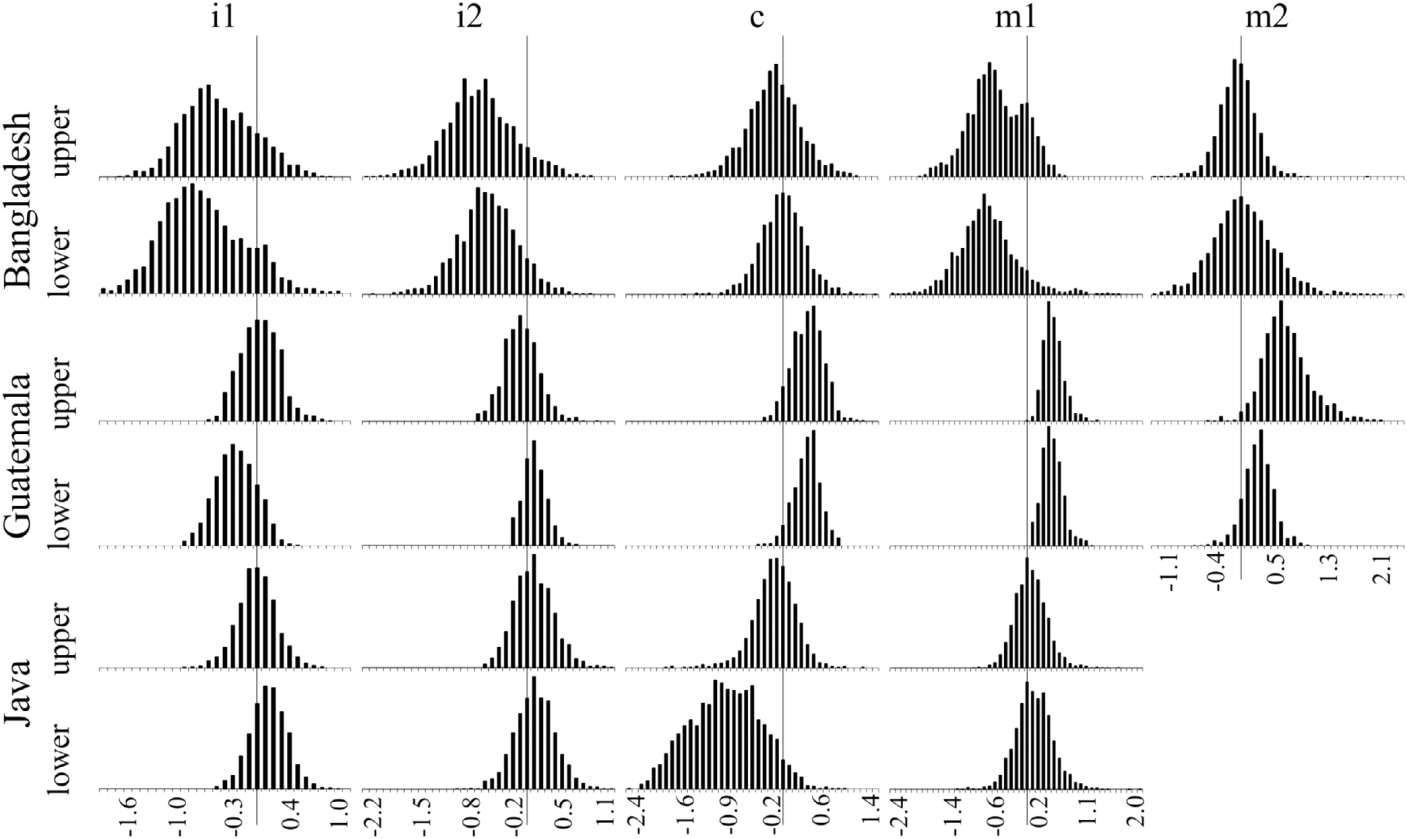
Distributions of *β*_died_ parameter estimates for each tooth (except the Javanese second molars) in the three samples from 2001 bootstrapped replicates. Negative values (a lower hazard) imply a later age at emergence in the mortality sample. Vertical lines pass through zero for each tooth.

Figure 1 shows distributions for the *β*_died_ coefficients for each tooth based on 2001 bootstrapped estimates. Distributions shifted to the left indicate that the mortality subsample exhibited delayed tooth emergence relative to the living subsample. Interestingly, the Bangladesh i^1^, i_1_, i^2^, i_2_, m^1^, and m_1_ are consistently shifted to the left by 1–2 months (Table 1). Although the observed shift was not significant for any tooth, it is suggestive of a bias in the mortality subsample that experienced a famine. In the chronically undernourished Javanese sample, there is no trend apparent, except for a slight trend toward delayed emergence in the lower canine. For the Guatemala mortality subsample, there is a clear trend toward advanced emergence in the canines, first molars and second molars of both jaws, although the differences are only significant for four teeth (c_1_¸ m^1^, m_1_, and m^2^). Interestingly, i_1_ hints at a delay in the mortality subsample.

Analysis of all samples simultaneously (Table 4), controlling for sex and with population‐specific fixed effects, showed no detectable differences between the living and mortality subsample, as the 95% confidence intervals for *β*_died_ always include zero. Because the Bangladesh and Java samples were similar in being chronically undernourished, a joint analysis of the Bangladesh and Java samples was undertaken (Table 5). No significant delays in the mortality subsample for any tooth were found. The effect sizes needed are between 2 weeks and a month for five teeth and 1–1.5 months for three teeth; delays greater than 3 months would be needed to detect bias in the second molars.

A more extensive analysis of the power to detect different effect sizes for the Bangladesh and Guatemala samples is given in the Supporting Information material (Tables S1 and S2).

## 3 Discussion

We assessed whether biological mortality bias exists for dental development in three non‐industrial populations by examining differences in emergence between surviving individuals and those who died. The three populations formed something of a natural experiment because of the different health conditions in each. Most of the Bangladesh children experienced chronic undernutrition and many also experienced a severe famine. Javanese children were from rural, poor households in an undernourished population, and the Guatemalan children were provided nutritional supplements and nutritional interventions when needed.

The only significant mortality bias was found for some teeth in the Guatemala sample where the posterior dentition was accelerated in the mortality subsample (Table 2). We have no obvious explanation for this unexpected finding, although the results may reflect differences in breastfeeding behavior and supplementation resulting from the nutritional intervention. Children supplemented at an earlier age are at an increased risk of morbidity and mortality because of the loss of passive immunity provided by breast milk and an increased exposure to food‐borne pathogens (Cunningham et al. 1991; Cunningham 1995; Hanson et al. 1994; Hanson 2000; Popkin et al. 1990). A related explanation is that the most well‐nourished mothers may have experienced, on average, an earlier return of ovulatory cycles postpartum. Some may have become pregnant while still breastfeeding, leading to an earlier weaning age. Additional research will be needed to uncover the cause and the role, if any, of the nutritional supplementation in the Guatemalan study on this paradoxical outcome, and whether the results hold more broadly.

In the pooled analyses, there were no significant differences found between the living and mortality subsamples, despite sufficient power to detect small differences in emergence timing (Table 4). Although this seems like an encouraging result, it is possible that opposing biases, particularly between the Bangladesh and Guatemala samples, canceled each other out, leaving no net effect. We tested this by an analysis of the pooled Bangladesh and Javanese samples (Table 5) and found no bias.

The net result is limited evidence for biological mortality bias in the timing of deciduous tooth emergence that arises under some circumstances as reflected in the posterior dentition for the Guatemala sample. In the two populations experiencing the greatest nutritional stress, we detected no significant biases, with enough power to find differences of under 1 month.

Our results are consistent with other studies investigating mortality bias in dental development. Spake et al. (2021) examined dental development stages in 206 children aged 12 years or younger at death. They classified deaths as natural or accidental to approximate “frailer” and “less frail” samples. They found no evidence of differences in stages between the two groups. Stull et al. (2021) used radiographic images from living and mortality samples and found no differences in dental development stages in 239 children aged 5–15. These two studies, combined with the present study, suggest that biological mortality bias is minimal in dental development and provides some justification for using these traits in reference samples.

Previous findings strongly suggest population differences in the timing of tooth emergence (e.g., Holman and Jones 1998, 2003; Muthu et al. 2024). The means in Tables 1, 2, 3 reiterate these findings and reinforce the need for population‐specific standards. Lampl and Johnston (1996) have demonstrated the need for population‐specific dental development standards for aging skeletons as well. Numerous studies have found that nutrition, health, and socioeconomic conditions can affect deciduous tooth emergence (Alvarez 1995; Bastos et al. 2007; Enwonwu 1973; Gaur and Kumar 2012; Holman and Yamaguchi 2005; Muthu et al. 2024; Möhlhenrich et al. 2023; Oziegbe et al. 2009; Rao Kodali et al. 1993; Strumpf et al. 2023; Truswell and Hansen 1973) and may lead to differences among populations with different levels of nutritional stress. Therefore, population‐specific reference samples should be used when possible, and the reference sample should come from a similar nutritional and disease ecology as the target sample. Since all three of our samples were in subtropical environments, our results may not apply to children in other contexts, such as arctic or temperate regions.

**TABLE 3:** Parameter estimates, mean emergence times, percent censored observations, and detectible effect sizes for the Javanese sample.

Tooth	Median parameter estimates (95% central confidence interval)				Mean emergence age (months)		Percent right censored		*N*_eff_	*E*_mo_
	*a*	*b*	*β*_sex_	*β*_died_	Alive	Died	Alive	Died		
i^1^	2.90 (2.87, 2.93)	0.112 (0.100, 0.125)	−0.262 (−0.438, −0.091)	−0.002 (−0.402, 0.406)	11.1	11.1	5.7	59.4	328	1.5
i^2^	3.00 (2.97, 3.03)	0.132 (0.118, 0.146)	−0.188 (−0.358, −0.015)	0.084 (−0.340, 0.567)	13.2	13.0	8.0	59.4	324	2.4
c^1^	3.34 (3.31, 3.38)	0.118 (0.104, 0.132)	0.005 (−0.193, 0.195)	−0.123 (−0.700, 0.365)	20.4	20.7	32.6	81.3	347	20
m^1^	3.25 (3.23, 3.28)	0.106 (0.097, 0.116)	0.203 (0.027, 0.389)	0.052 (−0.389, 0.557)	17.4	17.3	17.0	71.9	359	2.7
m^2^	3.62 (3.57, 3.68)	0.102 (0.085, 0.121)	0.173 (−0.212, 0.587)	—	28.7	—	83.7	100.0	94	7.2
i_1_	2.84 (2.81, 2.87)	0.123 (0.111, 0.135)	−0.294 (−0.464, −0.123)	0.140 (−0.235, 0.491)	10.4	10.0	5.0	53.1	—	—
i_2_	3.18 (3.14, 3.22)	0.153 (0.139, 0.169)	0.002 (−0.168, 0.172)	0.107 (−0.355, 0.584)	16.3	16.0	16.8	68.8	372	3.6
c_1_	3.38 (3.35, 3.42)	0.120 (0.106, 0.133)	−0.061 (−0.269, 0.147)	−0.931 (−2.03, 0.148)	22.1	25.8	43.7	93.8	317	20
m_1_	3.30 (3.28, 3.33)	0.111 (0.101, 0.122)	0.201 (0.025, 0.385)	0.114 (−0.412, 0.695)	18.7	18.5	22.7	75.0	359	8.9
m_2_	3.63 (3.58, 3.70)	0.115 (0.096, 0.134)	0.322 (−0.053, 0.730)	—	28.3	—	81.3	100.0	—	—

*Note:* A sample of 468 children of whom 48 died during the study. Median of 2001 bootstrapped replicates; 95% CIs for *β* parameters that include zero are not significant. *N*_eff_ is the effective sample size that contributed to estimates; *E*_mo_ is the effect size (months) that would have an 80% probability of being measured as statistically significant for the given *N*_eff_.

**TABLE 4:** Parameter estimates and detectible effect sizes from a combined analysis of the Bangladeshi, Javanese, and Guatemalan samples.

Tooth	Median parameter estimates (95% central confidence interval)						*N*_eff_	*E*_mo_
	*a*	*b*	*β*_sex_	*β*_Bangladesh_	*β*_Java_	*β*_died_		
i^1^	2.88 (2.86, 2.90)	0.114 (0.103, 0.126)	−0.188 (−0.317, −0.049)	−0.550 (−0.714, −0.369)	−0.259 (−0.406, −0.121)	−0.234 (−0.629, 0.170)	807	0.6
i^2^	2.92 (2.91, 2.95)	0.116 (0.106, 0.126)	−0.156 (−0.278, −0.021)	−0.875 (−1.048, −0.730)	−0.634 (−0.754, −0.499)	−0.253 (−0.632, 0.163)	851	0.6
c^1^	3.28 (3.26, 3.30)	0.100 (0.093, 0.108)	−0.159 (−0.268, −0.041)	−0.531 (−0.712, −0.377)	−0.403 (−0.516, −0.272)	0.054 (−0.316, 0.380)	962	0.8
m^1^	3.18 (3.16, 3.20)	0.098 (0.091, 0.104)	0.041 (−0.104, 0.210)	−0.030 (−0.277, 0.181)	−0.472 (−0.602, −0.345)	−0.151 (−0.605, 0.343)	780	0.9
m^2^	3.57 (3.55, 3.59)	0.113 (0.102, 0.125)	−0.014 (−0.149, 0.140)	−0.085 (−0.426, 0.250)	−0.479 (−0.715, −0.242)	0.222 (−2.62, 0.659)	631	2.5
i_1_	2.78 (2.76, 2.80)	0.120 (0.110, 0.132)	−0.085 (−0.202, 0.031)	−0.806 (−0.969, −0.655)	−0.710 (−0.854, −0.581)	−0.291 (−0.678, 0.135)	917	0.5
i_2_	3.07 (3.05, 3.09)	0.135 (0.126, 0.144)	−0.023 (−0.128, 0.092)	−0.676 (−0.854, −0.526)	−0.672 (−0.804, −0.562)	−0.116 (−0.421, 0.220)	1004	0.8
c_1_	3.31 (3.29, 3.33)	0.105 (0.096, 0.113)	−0.127 (−0.274, −0.005)	−0.688 (−0.916, −0.487)	−0.594 (−0.724, −0.432)	0.003 (−0.403, 0.525)	907	1.1
m_1_	3.24 (3.20, 3.27)	0.123 (0.093, 0.127)	0.120 (−0.082, 0.267)	−0.313 (−0.638, −0.027)	−0.430 (−0.697, −0.234)	−0.073 (−0.568, 0.473)	780	1.8
m_2_	3.56 (3.53, 3.58)	0.118 (0.104, 0.132)	0.030 (−0.148, 0.210)	−0.132 (−0.487, 0.156)	−0.528 (−0.731, −0.302)	0.013 (−0.506, 0.623)	427	2.5

*Note:* Median of 2001 bootstrapped replicates; 95% CIs for *β* parameters that include zero are not significant; *N*_eff_ is the effective sample size that contributed to estimates; *E*_mo_ is the effect size (months) that would have an 80% probability of being measured as statistically significant for the given *N*_eff_.

**TABLE 5:** Parameter estimates and detectible effect sizes from a combined analysis of the Bangladeshi and Javanese samples.

Tooth	Median parameter estimates (95% central confidence interval)					*N*_eff_	*E*_mo_
	*a*	*b*	*β*_sex_	*β*_Bangladesh_	*β*_died_		
i^1^	2.90 (2.87, 2.93)	0.122 (0.103, 0.142)	−0.214 (−0.423, −0.017)	−0.283 (−0.462, −0.102)	−0.345 (−0.881, 0.325)	525	0.6
i^2^	2.99 (2.96, 3.03)	0.134 (0.116, 0.150)	−0.206 (−0.387, −0.046)	−0.269 (−0.451, −0.073)	−0.367 (−0.953, 0.334)	502	0.7
c^1^	3.32 (3.30, 3.35)	0.112 (0.097, 0.125)	−0.121 (−0.305, 0.065)	−0.151 (−0.357, 0.054)	−0.117 (−0.647, 0.369)	485	0.9
m^1^	3.24 (3.21, 3.27)	0.115 (0.104, 0.128)	0.130 (−0.046, 0.319)	0.436 (0.168, 0.719)	−0.371 (−1.119, 0.300)	511	1.2
m^2^	3.63 (3.57, 3.73)	0.149 (0.117, 0.197)	0.063 (−0.236, 0.355)	0.439 (0.026, 0.857)	−0.032 (−0.602, 0.571)	169	3.8
i_1_	2.85 (2.82, 2.87)	0.127 (0.112, 0.140)	−0.240 (−0.403, −0.086)	−0.108 (−0.277, 0.061)	−0.364 (−0.912, 0.308)	532	0.5
i_2_	3.17 (3.14, 3.21)	0.156 (0.141, 0.173)	−0.017 (−0.178, 0.139)	−0.008 (−0.208, 0.177)	−0.289 (−0.811, 0.308)	554	1
c_1_	3.36 (3.32, 3.40)	0.116 (0.101, 0.132)	−0.221 (−0.469, 0.010)	−0.129 (−0.350, 0.121)	−0.264 (−0.858, 0.238)	423	1.2
m_1_	3.26 (3.23, 3.31)	0.110 (0.099, 0.121)	−0.040 (−0.275, 0.249)	0.111 (−0.173, 0.414)	−0.439 (−1.168, 0.422)	449	1.3
m_2_	3.63 (3.57, 3.70)	0.135 (0.107, 0.168)	0.237 (−0.079, 0.503)	0.186 (−0.177, 0.592)	0.005 (−0.938, 1.051)	173	3.3

*Note:* Median of 2001 bootstrapped replicates; 95% CIs for *β* parameters that include zero are not significant. *N*_eff_ is the effective sample size that contributed to estimates. *E*_mo_ is the effect size (months) that would have an 80% probability of being measured as statistically significant for the given *N*_eff_.

Our results may not generalize to other dental or skeletal traits used for studying growth. Deciduous tooth emergence has long been thought to be buffered against moderate nutritional stress relative to other skeletal traits (Jelliffe and Jelliffe 1973). Other traits may be more susceptible to biological mortality bias (Spake and Cardoso 2019), and they should be evaluated using living samples with a mortality subsample (Stull et al. 2021), from documented cemetery samples as suggested by Saunders and Hoppa (1993) or from other mortality samples with natural and accidental causes of death recorded (Spake and Cardoso 2019; Spake et al. 2021).

Attempts to assess biological mortality bias in traits associated with long bone growth have produced weak, mixed results. Spake and Cardoso (2019) used two different approaches to assess biological mortality bias in stature and found small biases. Stull et al. (2021) examined long bone growth from radiographic images from living and mortality samples and found almost no differences to suggest bias. They argue that in contemporary populations, at least, there is almost no evidence for systematic differences between a living sample and a mortality sample for long bone lengths and dental development.

We found no strong evidence of mortality bias in tooth emergence in the pooled analysis of nutritionally stressed samples. This finding may reflect that mortality acts fast enough in children that it leaves no effects on the timing of deciduous emergence. This pattern would be expected in ecological settings where infant and child mortality result from fast‐acting infectious diseases. In Bangladesh, respiratory infections and diarrheal disease were the major causes of death for infants and children who survive the neonatal period (ICDDR,B 1992). Jelliffe and Jelliffe (1973) surveyed the literature and concluded that most childhood illnesses do not delay dental emergence. Yet, diseases of childhood can result in stunted growth (Saunders and Hoppa 1993; King and Ulijaszek 1999). For example, diarrheal disease is associated with stunted growth in undernourished populations (Martorell et al. 1975; Lutter et al. 1992). However, the spectrum of childhood diseases and causes of death may differ by ecological context.

Longitudinal studies of growth and development in living populations have provided some of the best evidence for biological mortality bias for linear growth traits (reviewed in Saunders and Hoppa 1993). There are some difficulties, however, in using these studies for investigating biological mortality bias. The studies require relatively large numbers of participants because of the low level of infant and child mortality. Any future studies will require even larger samples because contemporary research ethics likely require health interventions for children clearly at risk of mortality. This is the reason there were no unsupplemented control villages in the Guatemalan study (Habicht and Martorell 1992). Such interventions may further obscure the presence of mortality bias. The alternative approaches outlined above may prove more fruitful in investigating biological mortality bias.

## Author Contributions

**Darryl J. Holman:** conceptualization, writing – original draft, methodology, formal analysis, project administration, investigation, writing – review and editing. **Chris J. Dolinski:** writing – review and editing, investigation, writing – original draft.

## Conflicts of Interest

The authors declare no conflicts of interest.

## Supporting information


**Table S1:** Sample sizes needed in the Bangladesh sample for detecting a difference between the living and mortality sample for effect sizes (delayed emergence) from 1 to 6 months.
**Table S2:** Sample sizes needed in the Guatemala sample for detecting a difference between the living and mortality sample for effect sizes (delayed emergence) from 1 to 6 months.

## Data Availability

Data sets and codebooks can be obtained from International Center for Diarrhoeal Disease Research, Bangladesh (ICDDR,B 1990), the Ngaglik Study Team (1978), and the Institute of Nutrition of Central America and Panama (Habicht and Martorell 1992).

## Endnotes

^1^
In addition to biological mortality, Saunders and Hoppa (1993) define two other types of mortality bias. Cultural mortality bias is variation in mortuary practice that leads to differential preservation of individuals or classes of individuals. Environmental mortality bias reflects differential effects of skeletal preservation resulting from environmental factors.

^2^
The survival function is the complement of the cumulative distribution function, *S*(*t*|*a*, *b*) = 1 − *F*(*t*|*a*, *b*). In this context “survival” does not refer to mortality of the child. Rather it refers to the absence of the event of interest (tooth emergence). This is standard statistical terminology, but can be confusing when one of the covariates is death of a child.
