# Supplementary material for: Assessing Biological Mortality Bias From Deciduous Tooth Emergence
Source: Am J Biol Anthropol. 2026 Jan 10;189(1):e70202. doi: 10.1002/ajpa.70202 (PMC12789964; doi:10.1002/ajpa.70202)
Supplement: Supplementary file 1 — Table S1: Sample sizes needed in the Bangladesh sample for detecting a difference between the living and mortality sample for effect sizes (delayed emergence) from 1 to 6 months. Table S2: Sample sizes needed in the Guatemala sample for detecting a difference between the living and mortality sample for effect sizes (delayed emergence) from 1 to 6 months. [file AJPA-189-e70202-s001.docx]

Table S1. Sample sizes needed in the Bangladesh sample for detecting a difference between the living and mortality sample for effect sizes (delayed emergence) from 1 to 6 months.

| Tooth | *N_eff_* | *N*_1_*_mo_* (β_1_*_mo_*) | *N*_2_*_mo_* (β_2_*_mo_*) | *N*_3_*_mo_* (β_3_*_mo_*) | *N*_4_*_mo_* (β_4_*_mo_*) | *N*_5_*_mo_* (β_5_*_mo_*) | *N*_6_*_mo_* (β_6_*_mo_*) |
| --- | --- | --- | --- | --- | --- | --- | --- |
| i^1^ | 197 | 2396 (-0.354) | 705 (-0.653) | 364 (-0.908) | 235 (-1.130) | 171 (-1.324) | 134 (-1.495) |
| i^2^ | 178 | 2975 (-0.327) | 865 (-0.607) | 443 (-0.849) | 284 (-1.060) | 205 (-1.247) | 160 (-1.413) |
| c^1^ | 138 | 1609 (-0.355) | 467 (-0.658) | 238 (-0.922) | 152 (-1.153) | 110 (-1.358) | 85 (-1.540) |
| m^1^ | 152 | 2008 (-0.380) | 589 (-0.702) | 304 (-0.977) | 196 (-1.217) | 142 (-1.427) | 111 (-1.613) |
| m^2^ | 75 | 1395 (-0.179) | 380 (-0.343) | 183 (-0.494) | 112 (-0.633) | 77 (-0.761) | 58 (-0.881) |
| i_1_ | 201 | 2268 (-0.420) | 681 (-0.765) | 358 (-1.055) | 235 (-1.303) | 174 (-1.516) | 138 (-1.703) |
| i_2_ | 182 | 3716 (-0.260) | 1053 (-0.489) | 526 (-0.691) | 331 (-0.872) | 235 (-1.035) | 180 (-1.182) |
| c_1_ | 106 | 1201 (-0.302) | 342 (-0.566) | 172 (-0.798) | 109 (-1.004) | 77 (-1.189) | 60 (-1.356) |
| m_1_ | 89 | 1337 (-0.396) | 393 (-0.731) | 203 (-1.016) | 131 (-1.264) | 96 (-1.480) | 75 (-1.672) |
| m_2_ | 60 | 2927 (-0.187) | 799 (-0.359) | 386 (-0.516) | 236 (-0.661) | 163 (-0.794) | 122 (-0.919) |

Symbols: *N_eff_* is the effective number of individuals for each tooth taken from Holman and Jones (1998, Table 5); *N*_1_*_mo_* is the effective number of individuals needed for an 80% probability of detecting a one-month difference between the living and mortality sample at α = 5%; β_1_*_mo_* is the coefficient value (β*_died_* in Table 1) that gives a one month delay in emergence.

Table S2. Sample sizes needed in the Guatemala sample for detecting a difference between the living and mortality sample for effect sizes (delayed emergence) from 1 to 6 months.

| Tooth | *N_eff_* | *N*_1_*_mo_* (β_1_*_mo_*) | *N*_2_*_mo_* (β_2_*_mo_*) | *N*_3_*_mo_* (β_3_*_mo_*) | *N*_4_*_mo_* (β_4_*_mo_*) | *N*_5_*_mo_* (β_5_*_mo_*) | *N*_6_*_mo_* (β_6_*_mo_*) |
| --- | --- | --- | --- | --- | --- | --- | --- |
| i^1^ | 282 | 597 (-0.463) | 181 (-0.840) | 96 (-1.153) | 64 (-1.418) | 47 (-1.646) | 38 (-1.844) |
| i^2^ | 348 | 997 (-0.419) | 299 (-0.766) | 157 (-1.057) | 103 (-1.306) | 76 (-1.522) | 60 (-1.711) |
| c^1^ | 477 | 1765 (-0.367) | 514 (-0.680) | 263 (-0.951) | 169 (-1.187) | 122 (-1.396) | 95 (-1.581) |
| m^1^ | 269 | 411 (-0.427) | 122 (-0.783) | 64 (-1.085) | 41 (-1.345) | 30 (-1.571) | 24 (-1.770) |
| m^2^ | 461 | 5775 (-0.284) | 1625 (-0.535) | 808 (-0.759) | 505 (-0.960) | 357 (-1.141) | 273 (-1.306) |
| i_1_ | 386 | 1002 (-0.432) | 304 (-0.783) | 161 (-1.076) | 107 (-1.324) | 79 (-1.537) | 63 (-1.723) |
| i_2_ | 449 | 930 (-0.335) | 271 (-0.620) | 138 (-0.867) | 89 (-1.083) | 64 (-1.274) | 50 (-1.444) |
| c_1_ | 484 | 1606 (-0.327) | 462 (-0.611) | 234 (-0.858) | 148 (-1.077) | 107 (-1.271) | 82 (-1.446) |
| m_1_ | 331 | 1034 (-0.309) | 296 (-0.578) | 150 (-0.813) | 95 (-1.021) | 68 (-1.207) | 52 (-1.373) |
| m_2_ | 255 | 1630 (-0.251) | 454 (-0.475) | 224 (-0.677) | 139 (-0.860) | 98 (-1.026) | 74 (-1.177) |

Symbols: *N_eff_* is the effective number of individuals for each tooth taken from Holman and Jones (1998, Table 4); *N*_1_*_mo_* is the effective number of individuals needed for an 80% probability of detecting a one-month difference between the living and mortality sample at α = 5%; β_1_*_mo_* is the coefficient value (β*_died_* in Table 2) that gives a one month delay in emergence.
